# Does It Feel Like Yesterday or Like It’s Been Forever? Subjective Time Since Sex in Romantic Relationships

**DOI:** 10.1177/19485506231217529

**Published:** 2023-12-24

**Authors:** Elysia Vaccarino, Stephanie Raposo, Amy Muise

**Affiliations:** 1 York University, Toronto, Ontario, Canada

**Keywords:** subjective time, sexuality, sexual frequency, relationship satisfaction, sexual satisfaction, romantic relationships

## Abstract

Sexual frequency in relationships is associated with satisfaction. Beyond objective reports, the subjective time since sex might also be associated with relationship evaluations. When sex feels further away, do people feel less satisfied? Do they desire sex more? In a cross-sectional study (Study 1), when one’s last sexual experience felt further away, people reported lower sexual satisfaction and desire. In an experimental study (Study 2), women (but not men) who were made to feel that the last sex was further (vs. closer) reported lower desire, but overall, there was limited evidence of causal effects. However, in a 21-day daily experience study (Study 3), within-person lagged models revealed that sex feeling further away was associated with lower sexual satisfaction, but higher desire, tomorrow, and higher desire and satisfaction were associated with sex feeling further away tomorrow. Subjective time since sex has nuanced associations in relationships, especially with desire and satisfaction in daily life.

Frequent sex in a romantic relationship—up to a frequency of once a week—is linked to relationship satisfaction and well-being (Muise et al., 2016). At the same time, sexual frequency tends to decline over time in a relationship and these declines can be associated with lower sexual and relationship satisfaction (McNulty et al., 2016). Beyond objective reports of how often a couple has sex, it is also possible that how long it *feels* between sexual experiences is linked to satisfaction. For one person, having engaged in sex 7 days ago might feel like a very recent experience, whereas for another person, a sexual encounter from 7 days ago could feel quite far away. In the current research, we draw on research and theory about subjective time (e.g., Cortes et al., 2017) to test how the subjective time since a person’s last sexual encounter is associated with sexual and relationship satisfaction and sexual desire.

## The Subjective Experience of Time

Although people often think about time as objective (e.g., “the drive will take us two hours”), it is also subjective (e.g., “it feels like we have been driving forever!”). Research demonstrates that perceptions of the temporal distance of events vary and can be malleable (e.g., Ross & Wilson, 2002), and these differences are associated with important outcomes. For example, when a transgression is perceived to be further away (vs. closer), people are more willing to forgive the transgressor (Wohl & McGrath, 2007). When used adaptively, subjective time estimates can help people minimize the consequences associated with past negative events (Ross & Wilson, 2002; Taylor, 1991) or be used as a tool for self-enhancement or self-protection (Peetz et al., 2009), by which negative events are pushed further away and successes or positive experiences are kept closer (Peetz & Wilson, 2008). Thus, previous work suggests that the temporal distance of previous experiences can vary and this variation has implications for well-being and motivation.

## Subjective Time in Romantic Relationships

The temporal distancing of relational events is associated with relationship quality. When negative relational memories feel subjectively closer than positive ones, people are more likely to “kitchen think” (i.e., ruminate about previous negative events during a conflict), which is associated with more intense conflict and poorer perceptions of the relationship (Cortes & Wilson, 2016). In addition, satisfied partners tend to keep happy relational events subjectively closer and negative events more distant, which is associated with higher relationship satisfaction (Cortes et al., 2017). It is possible then, that when important, positive interactions in relationships feel further away, this is associated with lower satisfaction.

One important and often positive event in a relationship is sex. People tend to report greater relationship satisfaction, positive affect, and well-being on days when they engage in sex with their partner compared with days when sex does not occur (Debrot et al., 2017; Kahneman et al., 2004; Kashdan et al., 2018), and engaging in frequent sex (up to a frequency of once a week) is associated with feeling more satisfied with one’s sex life, relationship, and life in general (Cheng & Smyth, 2015; Hicks et al., 2016; McNulty et al., 2016; Muise et al., 2016; Schoenfeld et al., 2017). Notably, the recency of sexual activity is also associated with satisfaction. After engaging in sex with a partner, sexual satisfaction, or sexual “afterglow,” has been found to remain for 48 hr, which in turn, is associated with greater relationship satisfaction (Meltzer et al., 2017). One reason for this sexual afterglow may be because sex feels closer in time, but this might dissipate as the sexual experience begins to feel further away. Therefore, beyond objective frequency, when sex feels subjectively further away, people might feel less satisfied.

However, the subjective time since sex might be differentially associated with sexual desire; when sex feels further away, people might desire it more. Research on the subjective duration of time has shown that when people are awaiting important information, time feels like it is moving more slowly (i.e., it is harder to wait; Rankin et al., 2019). In addition, perceptions of an event as closer in time can be associated with motivation (Peetz et al., 2009); inducing people to view the consequences of climate change as more proximal can enhance pro-environmental motivations (i.e., to engage in environmentally conscious behaviors; Bashir et al., 2014). Therefore, applied to the sexual domain, although sex feeling further away could be linked to lower satisfaction, it may also be associated with a higher desire for sex—a possibility we will explore in the current research.

## The Current Research

Across three studies, using cross-sectional (Study 1), experimental (Study 2), and dyadic daily experience methods (Study 3), we investigated whether the subjective time since a person’s last sexual encounter with their partner is associated with sexual and relationship satisfaction, as well as sexual desire. We expected that when the last sexual encounter feels farther away (vs. closer), people would report lower relationship and sexual satisfaction. We also explored associations with sexual desire, as sex feeling further away could be linked to lower desire, given the association between sexual desire and sexual and relationship satisfaction (e.g., Mark, 2012), or it could be associated with higher desire because it feels like it has been longer since sex occurred. We also tested whether the other associations remain significant when controlling for desire and explored gender differences. Data and code for all studies are available here: https://osf.io/zt9pe/?view_only=512a80bf9b2a424f9fdbd0f74b9b0fc0.

## Study 1

We conducted an initial, exploratory cross-sectional study to examine the associations between subjective time since sex and relationship satisfaction, sexual satisfaction, and sexual desire.

### Method

#### Participants and Design

We recruited people in relationships to complete an online survey via Prolific Academic. A power analysis with G*Power (Faul et al., 2007) using an estimate of the average effect size in social psychology *(f* = .20; Fraley & Vazire, 2014) indicated that 255 participants would be required to achieve 90% power. We recruited 266 participants to account for the removal of inattentive participants. After data exclusions, our final sample was 254 people in a committed intimate relationship of at least 12 months (see Table 1 for demographics).

**Table 1:** Demographics Across Studies

		Study 1		Study 2		Study 3	
Demographic Variable	Response Options	*N*	%	*N*	%	*N*	%
Gender	Men	104	40.9	115	47.5	349	50.4
	Women	147	57.9	124	51.2	340	49.1
	Non-binary	2	0.8	1	0.4		
	Missing	1	0.4	2	0.8	4	0.6
Age	*M, Med, SD, Range*	32.92, 31.00, 10.48, 49.00		32.63, 30.00, 10.17, 58.00		40.13, 38.00, 12.74, 58.00	
Sexual orientation	Heterosexual	221	87.0	192	81.4	618	89.2
	Bisexual	20	7.9	22	9.1	41	5.9
	Asexual			7	2.9	3	0.4
	Lesbian	4	1.6	6	2.5	7	1.0
	Pansexual	5	2.0	4	1.7	4	0.6
	Gay	1	0.4	2	0.8	11	1.6
	Queer	1	0.4	2	0.8	4	0.6
	Not listed	2	0.8	2	0.8	1	0.1
	Questioning					3	0.4
Ethnicity	White	204	80.3	159	65.7	613	88.5
	Black	4	1.6	11	4.5	14	2.0
	East Asian	14	5.5	20	8.3	13	1.9
	South Asian	4	1.6	18	7.4	26	3.8
	Southeast Asian			3	1.2		
	Latin American	9	3.5	11	4.5	6	0.9
	Bi- or multi-ethnic/racial	2	0.8	14	4.5	17	2.5
	Middle Eastern			4	1.7	1	0.1
	Ashkenazi Jewish			1	0.4		
	Native American/First Nation	12	4.7			2	0.3
	Not Listed					1	0.1
	Missing	5	2.0	1	0.4		
Marital status	Married	113	44.5	113	46.7	385	55.6
	Not married (e.g., living together, common law, dating, and engaged, not listed)	141	55.5	127	52.5	308	44.4
Relationship length (years)	*M, Med, SD, Range*	8.72, 5.00, 8.50, 52.00		8.50, 5.25, 8.39, 56.75		13.97, 10.63, 10.95, 53.17	

#### Measures

We assessed *subjective time since sex with* two items about how long it feels since the last sexual encounter with a partner: 1 = “Feels Very Close” to 10 = “Feels Very Distant” and 1 = “Feels Like Yesterday” to 10 = “Feels Like a Long Time Ago” (*r* = .95, *p <* .001; *M* = 3.28, *SD* = 2.44) and *objective time since sex* with the number of days since participant’s last sexual encounter with their partner (range, 0–365; *M* = 17.18, *SD* = 46.49). We assessed *relationship satisfaction* with the three-item subscale of the Perceived Relationship Quality Component (PRQC) inventory (Fletcher et al., 2000; e.g., “How satisfied are you with your relationship”) from 1 = “not at all” to 7 = “extremely” (α*=* .95, *M* = 5.55, *SD* = 1.26) *sexual satisfaction* with the 5-item GMSEX (MacNeil & Byers, 2005; e.g., “How would you describe your overall sexual relationship with your partner?”) rated on 7-point bipolar scales (e.g., 1 = “very negative” to 7 = “very positive”; α = .95, *M* = 5.55, *SD* = 1.26). *Sexual desire* was assessed using the 14-item Sexual Desire Inventory-2 (Spector et al., 1996; e.g., “During the last month, how often have you had sexual thoughts involving your partner?” from 0 = “Not at All” to 8 = “More Than Once a Day”; α = .84, *M* = 4.28, *SD* = 1.14).

### Analytic Approach

We conducted separate linear regression models to test the associations between subjective time since the last sexual encounter (controlling for the number of days since sex) and outcomes of interest. Predictor variables were centered around the sample mean. Correlations across key variables are reported in Table 2.

**Table 2:** Correlations Between Key Variables in Study 1

Variable	1	2	3	4	5
Subjective time since sex	—	.521**	−.485**	−.573**	–.222**
Actual days since sex		—	−.445**	−.444**	–.166**
Relationship satisfaction			—	.702**	.106
Sexual satisfaction				—	.300**
Sexual desire					—

*Note.* For partial correlations between subjective time, accounting for objective time, see Online Supplemental Materials (OSM).

** *p* < .001. *p < .05.

### Results

After accounting for the number of days since sex, in which more days since sex occurred was associated with lower relationship satisfaction (*b* = **−**.007, *p <* .001, 95% CI [−.010, −.004]) and sexual satisfaction (*b* = **−**.006, *p<*.001, 95% CI [−.009, −.002]), the subjective feeling of sex as farther away was associated with lower relationship satisfaction, *b* = **−**.183, *p <* .001, 95% CI [−.247, −.119], and sexual satisfaction, *b* = **−**.239, *p <* .001, 95% CI [−.300, −.178]. However, the association between subjective time since sex and relationship satisfaction was reduced to non-significance when controlling for sexual satisfaction. In addition, when sex felt further away, people reported lower sexual desire (*b* = **−**.087, *p* = .012, 95% CI [−.155, −.019]), and the associations with sexual and relationship satisfaction remained significant when sexual desire was controlled. Gender moderated the association between subjective time since sex and relationship satisfaction: while the association was significant for both men and women, it was stronger for men (see OSM).

### Brief Discussion

Study 1 provided initial evidence that when a person's last sexual experience *feels* further away people report lower sexual satisfaction and desire, above and beyond the objective time since sex. However, the association between subjective time and relationship satisfaction seemed to work through sexual satisfaction as it was non-significant when sexual satisfaction was controlled. Past research has found that time distortion, such as feeling like “time flies” during a task is associated with rating the task as more enjoyable (Sackett et al., 2010). Applied here, it is possible that when sex feels closer, people see it as more satisfying, and this might account for the association with relationship satisfaction.

## Study 2

In Study 2, we manipulated subjective time since one’s last sexual encounter to test effects on sexual and relationship satisfaction, and sexual desire. We also examined gender, given the results of a previous version of this study.^1^ This study was pre-registered on the OSF: https://osf.io/q6rnx.

### Method

#### Participants and Design

Participants were randomly assigned to one of two conditions (feels closer vs. feels farther; see Cortes et al., 2017). Given an estimated effect size of *f* = .16 from a previous study (see OSM), an a priori power analysis using G*Power (Faul et al., 2007) indicated that 720 participants would be required to achieve 99% power. We oversampled to allow for exclusions and recruited 801 participants through Prolific Academic. Participants had to be at least 18 years old and in a sexually active romantic relationship for at least 1 year. After exclusions (see OSM), our sample included 693 participants, just under our target sample size (see Table 1 for demographics).

#### Procedure

To manipulate the subjective time since sex, participants were asked to place on a slider scale when their last sexual encounter with their partner took place (adapted from Cortes et al., 2017). In the “Feels Close” condition, participants were asked to place the event on a slider scale that spanned from “Beginning of the relationship” to “Today,” while in the “Feels Far” condition, the slider scale spanned from “1 month ago” to “Today.” With the wider time frame in the “Feels Close” condition, the slider would be placed closer to “Today” making the participants’ last sexual encounter feel relatively close, compared with the shorter time frame of the “Feels Far” condition (see OSM). Following the manipulation, participants responded to two face-valid items assessing relationship (*M* = 6.04, *SD* = 1.07) and sexual satisfaction (*M* = 5.32, *SD* = 1.45), from 1 = “Not at all” to 7 = “Extremely,” and one item about their current desire to engage in sex with their partner on a scale from 1 = “Very Low or None at All” to 7 = “Very High” (*M* = 3.17, *SD* = 1.04). Participants then reported the number of days since their last sexual encounter with their partner (*M* = 8.42, *SD* = 8.78) and the quality of this last sexual encounter (*M* = 5.59, *SD* = 1.30).

### Analytic Approach

We conducted separate analyses of variance (ANOVAs) examining the effect of condition, and gender, controlling for the number of days since, on relationship satisfaction, sexual satisfaction, and sexual desire.

### Results

Participants in the feels close condition (*M* = 3.21, *SD* = 2.14) felt that their last sexual encounter was closer than those in the feels far condition (*M* = 4.11, *SD* = 2.56), controlling for actual days since sex, *F*(2,689) = 20.75, *p* < .001, η_p_^2^ = .029. However, there were few effects of condition on our key outcomes. There were no main effects of condition or gender, or an interaction between condition and gender, on relationship satisfaction (subjective time *p* = .985; gender *p* = .745; two-way interaction *p* = .127) or sexual satisfaction (subjective time *p* = .832; gender *p* = .985; two-way interaction *p* = .171). Results revealed no main effect of condition (*p* = .274) on sexual desire; however, there was a main effect of gender such that men tended to have higher sexual desire than women, *F*(4,684) = 70.13, *p* < .001, η_p_^2^ = .093, and a significant 2-way condition x gender interaction, *F*(4,684) = 3.96, *p* = .047, η_p_^2^ = .006. Simple effect analyses reveal that women felt less sexual desire when their last sexual experience was made to feel further away (*M* = 2.64, *SD* = 1.02) versus closer (*M* = 2.89, *SD* = 1.06), *F*(1,684) = 4.68, *p* = .031, η_p_^2^ = .007, but there was no effect for men, *F*(1,684) = .40, *p* = .527, η_p_^2^ = .001. The effect of condition on desire for women remained significant when the quality of last sexual encounter was controlled.

Given that most of the effects in our experimental study were not statistically significant, we re-ran the analyses as Bayes ANOVAs, which calculate a Bayes Factor, to evaluate the evidence in favor of the null.^2^ For the main effects of condition on key outcomes (sexual satisfaction, relationship satisfaction, sexual desire), the Bayes Factor was close to 0 and indicated substantial evidence for the null (.036–.089). In one case, the effect of condition on sexual desire for women (significant effect above), the Bayes Factor was over 1, which indicated evidence in favor of the alternative hypothesis (1.464), but not conclusive or strong evidence for the alternative (a Bayes Factor over 3 would be good evidence).

### Brief Discussion

Overall, the results of the first two studies provide inconsistent evidence about the association between subjective time since sex and satisfaction and desire. In Study 1, correlational evidence suggests that when sex feels subjectively further away, people feel less sexually satisfied and report lower sexual desire. In Study 2, our manipulation did make people feel like their last sexual experience was further (relative to closer) in time, but, overall, this does not causally impact our key outcomes. It is possible that there is not a causal effect, and any associations are due to additional variables. It is also possible that the manipulation did not affect sexual and relationship satisfaction because it was not overly relevant to people’s actual lives or did not reflect how they naturally experience subjective time since sex, and it is difficult to override people’s existing relationship evaluations (as they can be quite meaningful; see Park et al., 2021 for a discussion of challenges with brief manipulations).

## Study 3

To extend our previous studies, in Study 3, we conducted a 21-day daily experience study in which both partners could report on their sexual experiences each day and provide repeated assessments over time. One key advantage of this type of data is that it allows us to explore the direction of the associations in a more ecologically valid way by testing lagged day models. In Study 3, we also obtained a more accurate objective assessment of sexual frequency because we could non-intrusively calculate the number of days since participants last reported sex. Having reports from both partners also provided two informants of the couples’ objective sexual frequency and allowed us to examine whether a person’s perceptions of time since sex were associated with their partner’s relationship and sexual outcomes as well as their own.

### Method

#### Participants and Design

Participants were recruited through online (e.g., Craigslist) and physical (e.g., university campuses) advertisements in Canada and the United States. Eligible couples were living together or seeing each other at least 5 days per week, sexually active, 18 years of age or older, residing in Canada or the United States, able to read and understand English, and had daily access to a computer with internet. Both partners had to agree to participate. Our final sample consisted of 242 participants (121 couples) who provided 4,488 daily reports. A sensitivity analysis, correcting for the non-independence in the data (Wiley & Wiley, 2019), using G*power (Faul et al., 2007) indicated that a sample of 121 couples allowed for the detection of a minimum unstandardized slope of .29 for the association between subjective time since sex and sexual satisfaction (intraclass correlation = .13) with 80% power and a (two-sided) alpha of .05.

#### Procedure

Each partner completed a 60-minute online background survey, followed by 10- to 15-minute online surveys for 21 consecutive days, and a 20-minute follow-up survey 3 months later (see OSM for follow-up results). Each partner was compensated up to CAD 60. For the daily measures, we often used brief versions of the measures to increase efficiency and minimize participant attrition (Bolger et al., 2003). We assessed *subjective time since sex* each day with the same two items as Study 1 (*M=*3.68, *SD=*2.89). We accounted for *objective time since sex* in two ways. First, we calculated a “days since sex variable” using participants’ daily reports of whether they engaged in sex with their partner (i.e., yes or no). If they had sex that day, the value was 0, otherwise, it represented how many days it had been since they last reported sex. If a participant had missing data due to not completing the daily survey that day, but their partner had a response, we used the partner's report of sex (i.e., yes or no) on those missing days. After replacing values, 6.6% of the days were missing reports. Second, we calculated an aggregate of the number of times participants reported sex over the course of the daily experience study (*M* = 4.59, *SD* = 3.13). Each day, we asked participants about their *relationship satisfaction* with one item adapted from the PRQC but asked about that day (Fletcher et al., 2000; *M* = 5.98, *SD* = 1.31), *sexual satisfaction* using the same scale as in Study 1 but asked about that day (*Rc* = .96, *M* = 5.30, *SD* = 1.77), and *sexual desire* with the following item: “Today, I felt a great deal of sexual desire for my partner” from 1 = “Strongly Disagree” to 7 = “Strongly Agree” (*M* = 4.73, *SD* = 1.84).

### Analytic Approach

We tested two-level cross-classified models using mixed models in SPSS to account for the non-independence of partners within dyads and days. We modeled separate random intercepts for each partner within the dyad and treated the partners as indistinguishable and utilized compound symmetry matrices for the random effects to constrain the two partners to have the same parameters. Random slopes were tested for time-varying predictors, but models either failed to converge or random variances were unable to be computed, so we removed the random slopes. The fixed effects estimates changed negligibly between models with and without random slopes.

Our models were guided by the Actor Partner Interdependence Model (APIM; Kenny et al., 2006). We included both the aggregated (mean over the course of the daily diary) and person mean centered (centered around each person’s own average daily level) versions of subjective time since sex, although we only report the within-person effects, as this is the novel component of this study. In the same-day models, we controlled for sexual or relationship satisfaction on the previous day to rule out that the effects could be attributed to the previous day’s satisfaction. In subsequent analyses, we tested lagged effects in our predicted direction and the reverse direction. Correlations between key variables are reported in Table 3 and partners were correlated (*r* = .53; *p* < .001) on their reports of subjective time since sex.

**Table 3:** Correlations Across Key Variables in Study 3

Variable	1	2	3	4	5
1. Subjective time since sex	—	−.492**	−.311**	−.424**	−.406**
2. Sexual frequency		—	.164*	.211**	.286**
3. Relationship satisfaction			—	.539**	.414**
4. Sexual satisfaction				—	.449**
5. Sexual desire					—

*Note.* Variables are aggregates across the daily study and based on the overall individual sample of *N* = 242.

** *p* < .001. *p < .05.

### Same-Day Effects

In all models, we accounted for the number of days since sex, which was not associated with relationship satisfaction, *b* < .01, *SE* = .01, *t* = .01, *p* = 1.000, or sexual satisfaction, *b* = .01, *SE* = .01, *t* = .35, *p* = .177. Consistent with the findings from Study 1, on days when a person reported that their last sexual encounter felt farther away (vs. closer), they reported lower relationship satisfaction, *b* = −.10, *SE* = .01, *t* = −7.82, *p* < .001, but their partner did not report lower relationship satisfaction, *b <* .01, *SE* = .01, *t* = .04, *p* = .972, and both partners reported lower sexual satisfaction (own: *b* = −.19, *SE* = .01, *t* = −13.40, *p* < .001; partner: *b* = −.04, *SE* = .01, *t* = −2.65, *p* = .008). But, unlike Study 1, when predicting relationship satisfaction, the association held when controlling for sexual satisfaction. Consistent with Study 1 and the Study 2 findings for women, on days when sex felt further away, both partners reported lower sexual desire (own: *b* = −.15, *SE* = .02, *t* = −7.00, *p < .*001; partner: *b* = −.06, *SE* = .02, *t* = −2.93, *p = .*003). The other associations held controlling for daily sexual desire with one exception: the association between subjective time since sex and a partner’s sexual satisfaction was reduced to non-significance (*b* = −.02, *SE* = .13, *t* = −1.69, *p* = .092). In addition, in two cases the daily associations were stronger for men than women, but significant for both (see OSM).

### Lagged Day Effects

Finally, we ran lagged day models in which subjective time since sex today predicted the outcome tomorrow, controlling for the outcome today and the reverse. There were no significant associations between subjective time today and relationship satisfaction tomorrow (*b* = −.02, *SE* = .01, *t* = −1.46, *p* = .14) or the reverse (*b* = .02, *SE* = .06, *t* = .39, *p* = .70). Subjective time since sex today was associated with lower sexual satisfaction the next day (*b* = −.04, *SE* = .02, *t* = −2.61, *p = .*009), and on days when people felt more sexually satisfied, they felt like their last sexual experience was further away the next day (*b* = .34, *SE* = .05, *t* = 6.38, *p < .*001). In contrast to the same-day associations, on days when the last sexual experience felt further away, people reported *higher* sexual desire the next day (*b* = .09, *SE* = .02, *t* = 5.23, *p < .*001), and on days when desire was higher than typical, sex felt further away the next day (*b* = .32, *SE* = .04, *t* = 8.84, *p < .*001).

### Brief Discussion

The findings from Study 3 suggest a nuanced association between subjective time since sex and sexual satisfaction and desire, particularly when considering lagged (next day) effects. Although perceiving sex as further away today was associated with lower sexual satisfaction and desire that day and lower sexual satisfaction the next day, it was associated with higher sexual desire the next day. Perhaps when sex feels farther away, people might feel that their sex life is less satisfying, but they also might desire sex more. Also, when people were more sexually satisfied and had higher sexual desire, they felt like sex was farther away the next day, suggesting that when satisfied and interested in sex, it might feel like sex is harder to wait for (farther away).

## General Discussion

In the current set of studies, we tested how subjective time since sex is associated with satisfaction and desire. Our cross-sectional data (Study 1) demonstrated that people who felt their last sexual experience was farther away (vs. closer) reported lower relationship and sexual satisfaction, and lower sexual desire, but there was little evidence of causal effects (Study 2). However, Study 3, a daily experience study, revealed nuance in these associations. The same-day within-person associations between subjective time since sex and satisfaction and desire were consistent with the between-person findings in Study 1, but lagged-day analyses revealed interesting bi-directional associations. On days when people felt that sex was further away, they were less sexually satisfied the next day but also had higher desire. On days when people felt more sexually satisfied and had higher desire, they felt that sex was further away the next day, perhaps suggesting that when happy with your sex life, it is harder to wait for sex and thus, it feels farther away.

### Theoretical Implications

Although past research has examined how subjective time perspectives can be adaptive or maladaptive within romantic relationships (Cortes et al., 2017), we extended this literature by examining a specific positive relationship event—sex—and testing our effects across cross-sectional, experimental, and daily experience data given that most past work on subjective time has been assessed at one specific time point in the future or past (e.g., Cortes et al., 2017; Cortes & Wilson, 2016; Peetz et al., 2009; Ross & Wilson, 2002). Our cross-sectional and same-day associations suggest that when a person’s last sexual experience feels farther away (vs. closer), they report lower satisfaction and desire, which is consistent with past research demonstrating that people report higher relationship satisfaction when positive events feel closer (Cortes et al., 2017). However, given that sex is a regular occurrence in most relationships (Muise et al., 2016), in Study 3, we were able to assess sexual experiences repeatedly over time to test both how subjective time since sex is associated with satisfaction and desire the next day, and vice versa. These analyses revealed that although lower sexual satisfaction from sex feeling subjectively further away carried over to the next day (providing support for the proposed direction of effects), people also felt higher desire the next day. And, on days when people felt higher desire and sexual satisfaction, sex felt further away the next day. Past work shows that when waiting for potential bad news (e.g., exam results, medical test results), time feels like it is moving more slowly, and this is bi-directionally linked to distress; when you are more distressed, time moves more slowly, and when the time feels like it is moving more slowly, you feel more distressed (Rankin et al., 2019). In the current work, we uncovered a case in which temporal distance may increase anticipation for a (typically) positive event via enhanced desire, and that when people feel good about their sex life, sex might feel farther away because of the anticipation. The role of subjective duration of time in these findings is a key direction for future research.

Subjective time since sex was more robustly associated with sexual satisfaction compared to relationship satisfaction. Controlling for sexual satisfaction in Study 1 reduced the association between subjective time since sex and relationship satisfaction to non-significance, and even though in Study 3, greater subjective time since sex was associated with lower sexual satisfaction the next day, there was no lagged day effect on relationship satisfaction. Sexual satisfaction may be more proximal than relationship satisfaction and any associations between subjective time since sex and relationship satisfaction might work through sexual satisfaction. This is consistent with past research demonstrating that the enjoyment of a task is associated with differences in the subjective experience of time; when time feels like it is moving quickly, people tend to enjoy experiences more (Sackett et al., 2010). It is possible that when sex feels closer (vs. farther away), people recall the enjoyment of the sexual experience more and rate their sex life as satisfying, which could in turn be associated with overall relationship satisfaction in some cases.

### Limitations, Future Directions, Conclusions

Given the nuanced associations identified in the current research between subjective time since sex and satisfaction and desire, a key next step in the line of research is to consider when and for whom subjective time since sex is associated with higher (vs. lower) desire and satisfaction. Here, we have identified interesting bidirectional associations as well as differential links with satisfaction and desire in some cases. That is, the negative association between subjective time since sex and sexual satisfaction could reflect that when sex feels further away, people appraise it less positively or it could reflect that when people feel satisfied with their sex lives, sex feels frther away because they are anticipating the next encounter (i.e., people are more eager to repeat a positive experience). In addition, although seeing one’s last sexual experience as farther away is associated with lower sexual satisfaction the next day, it is also associated with higher desire. This suggests that although feeling like it has been a while since sex occurred can be linked to a less positive appraisal of one’s sex life, it might also be linked to a greater desire to engage in sex, perhaps to improve one’s sex life.

It is important to note, however, that we did not find causal evidence for our key associations, suggesting that the links between subjective time since sex and satisfaction and desire may not be causal, but instead attributed to other variables. Previous research has shown that how people attribute the subjective experience of time (i.e., why they feel like time is moving more quickly or slowly) can moderate or account for the association between subjective time and appraisal of an event (Sackett et al., 2010). Applied to the current findings, the associations between subjective time since sex and satisfaction and desire might differ based on (or be accounted for by) the reasons people attribute to the time since sex. In our experimental study, although people were made to feel that sex was further away (compared with the control group) they may have been better able to make an external attribution for the time since sex (i.e., they have not had privacy, they have been busy) than they would make in daily life (i.e., might be more attributed to their partner or relationship). In relationships, sex is a dyadic process and relies on both partners’ interest. Therefore, if a person attributes a longer time since sex to something they have little control over, such as their partner’s lack of interest or availability, they might feel less satisfied with their sex life. But, if they feel they have more control over when they might have sex next (i.e., their partner tends to be interested, they have a lot of uninterrupted time as a couple), feeling like the last experience is further away might boost their desire for sex and motivate them to pursue sex with their partner. These are possibilities to test in future research.

In terms of implications for romantic relationships, by investigating the subjective nature of our time perceptions, we can develop a fuller picture of how sex is associated with relationships and well-being. Over time in relationships, sexual frequency tends to decline (McNulty et al., 2016), but, despite this trend, it is possible that the benefits of engaging in sex could last longer if people can keep these experiences feeling close. Past research suggests that, on average, sexual satisfaction is heightened for 48 hours after sex (Meltzer et al., 2017), and this pattern is associated with greater relationship satisfaction. Future research might consider how couples can extend this sexual afterglow. Savoring positive experiences can intensify and prolong their benefits (Bryant & Veroff, 2007; Jose et al., 2012) and it is possible that savoring sexual experiences could have similar positive consequences, in part because savoring sex might make it feel subjectively closer.

Despite the limitations of the current work and many questions left to explore, the findings demonstrate that people’s perceptions of time since sex are subjective and that feeling that a recent sexual experience is closer versus farther away can have implications for satisfaction and desire. Future work can consider moderators of these associations as well as how to optimize perceptions of time in a way that is adaptive to people’s sexual and relational well-being.

## Supplemental Material

sj-docx-1-spp-10.1177_19485506231217529 – Supplemental material for Does It Feel Like Yesterday or Like It’s Been Forever?: Subjective Time Since Sex in Romantic Relationships Supplemental material, sj-docx-1-spp-10.1177_19485506231217529 for Does It Feel Like Yesterday or Like It’s Been Forever?: Subjective Time Since Sex in Romantic Relationships by Elysia Vaccarino, Stephanie Raposo and Amy Muise in Social Psychological and Personality Science

## Notes

1. This study is a revised version of a previous study in which the manipulation was not successful (non-significant manipulation check), possibly due to low power. Full details and results of this previous study are available in the Online Supplementary Materials (OSM).

2. This analysis was not pre-registered but requested during the review process.
