# Supplementary material for: Does It Feel Like Yesterday or Like It’s Been Forever? Subjective Time Since Sex in Romantic Relationships
Source: Soc Psychol Personal Sci. 2023 Dec 24;16(3):324–32. doi: 10.1177/19485506231217529 (PMC11805674; doi:10.1177/19485506231217529)
Supplement: sj-docx-1-spp-10.1177_19485506231217529 – Supplemental material for Does It Feel Like Yesterday or Like It’s Been Forever?: Subjective Time Since Sex in Romantic Relationships [file sj-docx-1-spp-10.1177_19485506231217529.docx]

**Study 1**

**Additional Outcome**

Participants were also asked how motivated they were to engage in a series of ***positive relationship behaviors*** towards their partner, such as “Show support for their interests or projects” and “Spend quality romantic time with them” on a scale from 1=“Not at all” to 7=“Very much” (adapted from Maxwell et al., 2019; α =.96, *M*=6.05, *SD*=.89). We did not report these effects in the main paper due to space considerations. After accounting for the number of days since sex, in which more days since sex occurred was associated with lower pro-relationship motivation (*b***=-**.004, *p=*.002, 95% CI [-.006, -.001]), the subjective feeling of sex as farther away was also associated with lower pro-relationship motivation, *b=***-**.119, *p<*.001, 95% CI [-.166, -.072]. However, as with relationship satisfaction (reported in the main paper), the effects remained significant when controlling for sexual desire.

**Partial Correlations Controlling for Objective Time**

In Study 1, at the request of a reviewers, we ran partial correlations controlling for objective time since sex (see Table S1). In Study 1, with one exception, all correlations remain significant (albeit attenuated) when partially controlling for the objective number of days since the last sexual encounter. The exception is that the correlation between pro-relationship motivation (an outcome reported above and not in the main text) and sexual desire was reduced to non-significant.

*Table S1: Correlations Between Key Variables in Study 1, Accounting for Objective Days Since Last Sexual Encounter*

| Variable | 1 | 2 | 3 | 4 | 5 |
| --- | --- | --- | --- | --- | --- |
| 1. Subjective Time since sex | - | -.301** | -.337** | -.439** | -.158* |
| 1. Pro-relationship motivation |  | - | .612** | .606** | **.104** |
| 1. Relationship satisfaction |  |  | - | .642** | .057 |
| 1. Sexual satisfaction |  |  |  | - | .254** |
| 1. Sexual desire |  |  |  |  | - |

*** p<.001; * p<.05. Bolded font indicates a correlation that became non-significant after accounting for objective days since sex.*

**Auxiliary Analyses**

Although, associations between sexual frequency and relationship satisfaction have been shown to be consistent across men and women (Muise et al., 2016), some research has found gender differences in how sexual aspects of the relationship are associated with relationship satisfaction (McNulty et al., 2016). Therefore, across studies we tested whether any of the associations were moderated by gender. In fact, in Study 1, gender (-1 = men; 1 = women) moderated one of the associations between subjective time since sex and relationship satisfaction, *b* **=** .073, *p =* .028, 95% CI [.008, .138]. Feeling that the most recent sexual experience was further away versus closer was associated with lower relationship satisfaction for both men (*b =* -.255, *p* < .001, 95% CI [-.362, -.148]) and women (*b =* -.109, *p* = .008, 95% CI [-.189 - .029]), but the effect was stronger for men.

In addition, in response to a reviewer’s comment, we ran models controlling for sexual satisfaction (grand mean centered) when predicting relationship satisfaction and motivation. In these models, the effects of subjective time on relationship satisfaction (*b =* -.04, *p* =.208, 95% CI [-.095, .021]) and pro-relationship motivation (*b =* -.02, *p* =.446, 95% CI [-.061, .027]) were reduced to non-significant when controlling for sexual satisfaction. In contrast, while the negative effect of objective time (i.e., the actual number of days since participants’ last sexual encounter) on relationship satisfaction remained significant (*b =* -.004, *p* =.010, 95% CI [-.006 -.001]), its effect on pro-relationship motivation was reduced to non-significant (*b =* -.001, *p* =.166, 95% CI [-.004, .001]). This means that the effects of subjective time since the last sexual encounter – and partly the effect of objective time since the last sexual encounter – were driven by overall levels of sexual satisfaction in Study 1.

**Study 2**

**Pilot Experimental Study**

In an initial experimental study, we used an experimental paradigm to test whether manipulating subjective time (how long it *feels*: further vs. closer) since one’s last sexual encounter predicts relationship and sexual satisfaction, and pro-relationship motivations (including another motivation measure: approach and avoidance relationship goals to assess general relationship motivation in addition to specific behaviors). This study was pre-registered on the OSF: <https://osf.io/fb4ae>

**Method**

***Participants and Design***

Participants were randomly assigned to one of two conditions (feels closer vs. feels farther) adapted from a paradigm used in previous research on subjective time (Cortes et al., 2017). With this design, an a priori power analysis using G*Power (Faul, et al., 2007) and an effect size of *f*=.20 indicated that 199 participants would be required to achieve 80% power. We oversampled by 15% to account for removal of participants who fail attention checks; thus, we recruited 233 participants through Prolific Academic. Participants had to be at least 18 years old and in a sexually active romantic relationship for at least one year. Two participants were removed for failing an attention check, eight participants were removed for participating from an ineligible country, 49 participants were removed due to significant COVID impact on themselves or their partners, five participants were removed for reporting their last sexual encounter with their partner as being over 100 days ago, one participant was removed for incomplete responses, and three participants did not disclose their gender and were excluded from analyses, leaving a sample of 165 participants. Given that our a priori power analysis required a larger sample than we achieved following exclusions, and did not account for testing gender interactions, we conducted a sensitivity analysis using G*Power (Faul et al., 2007), which found that our final sample could detect small effects of *f=*0.25 for the gender x subjective time interaction (power=.90, α=.05).

***Procedure***

Prior to the manipulation, participants reported the number of days since their last sexual encounter with their partner (*M=*9.64, *SD*=17.31). We controlled for their objective reports in the analyses. Participants were then randomly assigned to one of two subjective time conditions about their last sexual experience designed to manipulate whether this event felt further or closer to the present (adapted from Cortes et al., 2017). Participants were asked to place on a slider scale when their last sexual encounter within their relationship took place, and to manipulate subjective time we changed the origin label of the slider scale across conditions. In the “Feels Close” condition, participants were asked to place the event on a slider scale that spanned from “Beginning of the relationship” to “Today”, while in the “Feels Far” condition, the slider scale spanned from “1 month ago” to “Today”. With the wider time frame in the “Feels Close” condition, the slider would be placed closer to “Today” making the participants’ last sexual encounter feel relatively close, compared to the shorter time frame of the “Feels Far” condition, which should lead participants to feel that the last sexual encounter is further away given that the reference point is not as temporally distant (see the OSM for details of our pilot study and manipulation check).

Following the manipulation, participants responded to two face valid items assessing relationship (*M=*5.84, *SD*=1.14) and sexual satisfaction (*M=*5.17, *SD*=1.56), from 1=“Not at all” to 7=“Extremely,” and the same measure of pro-relationship motivations in Study 1, *α*=.95, *M*=5.84, *SD*=.91). As an additional measure of motivation, participants also reported on their approach and avoidance relationship goals from 1=“Not at all” to 7=“Extremely” with items such as “I will be trying to deepen my relationships with my romantic partner” (approach goals: 4-items; *α*=.87, *M*=5.78, *SD*=.96) and “I will be trying to stay away from situations that could harm my romantic relationship” (avoidance goals 4-items; *α*=.47, *M*=5.20, *SD*=.83) (adapted from Gable, 2006; Impett et al., 2008).

***Analytic Approach***

We conducted separate ANOVAs examining the effect of condition, controlling for number of days reported since last sexual encounter, on relationship satisfaction, sexual satisfaction, pro-relationship motivations, and relationship goals (approach and avoidance). Because critical gender differences were detected, we tested and will report gender differences in the models (0=men; 1=women).

**Results**

There were no main effects of subjective time (*p*=.843) or gender (*p*=.549) on relationship satisfaction; however, the interaction between condition and gender was significant, *F* (1, 160)=4.32, *p*=.039, *η_p_^2^*^=^.026. Simple effects, however, were not significant for women, *F*(1, 160)=2.54, *p=*.113 *η_p_^2^*^=^.016, or men, *F*(1, 160)=1.86, *p*=.175, *η_p_^2^*^=^.011. There was no main effect of subjective time (*p*=.374) or gender (*p=*.977), or an interaction between gender and subjective time, on sexual satisfaction (*p*=.737). There was also no main effect of subjective time (*p*=.938) or gender (*p=*.080), or an interaction between gender and subjective time, on pro-relationship motivations (*p*=.407).

Results revealed no main effect of subjective time (*p*=.548) on approach relationship goals. There was a main effect of participant gender on approach relationship goals (*p=*.006, η_p_^2=^.047), such that women (*M=*5.97, *SD=*.97) tended to report higher relationship approach goals overall compared to men (*M=*5.60, *SD=*.94). The interaction between gender and subjective time on relationship approach goals was significant *F*(1,159)=6.43, *p*=.012, *η_p_^2^*^=^.039. For men, when sex was made to feel farther away (*M=*5.83, *SD=*.93), they reported more approach-motivated relationship goals, *F*(1, 159)=5.18, *p=*.024, *η_p_^2=^*.032, than when sex was made to feel close (*M=*5.33, *SD=*.88). However, the subjective time condition did not predict approach relationship goals among women, *F*(1, 159)=1.84, *p*=.176, *η_p_^2=^*.011. There was no main effect of subjective time (*p*=.291) or gender (*p=*.103), or an interaction between gender and subjective time, on relationship avoidance goals (*p*=.884).

**Manipulation Check**

Prior to conducting this experimental study, we conducted a pilot test of the manipulation on Prolific Academic (*N* = 95). In particular, following the subjective time manipulation as described in the main text, participants were asked to respond on a 100-point slider scale across two items how long ago it felt since their last sexual encounter with their partner (from “Feels Very Close” to “Feels Very Distant”; and “Feels like Yesterday” to “Feel Like a Long Time Ago”; respectively). Results of this study confirmed that the manipulation was able to affect subjective time perceptions on the subsequent manipulation test, *F* (1, 92) = 6.97, *p* = .010). Specifically, participants in the “Feels Far” condition (*M* = 33.98; *SD* = 27.32) reported that their sexual encounter felt further away than those in the “Feels Close” condition (*M*  = 25.14; *SD* = 27.36).

In this study, following the manipulation, participants were asked to respond to this same question: “How long ago does it feel since your last sexual encounter with your partner?” however, instead of a slider scale, responses were measured on two separate items with scales from 1 (Feels Very Close) to 10 (Feels Very Distant), and 1 (Feels Like Yesterday) to 10 (Feels Like A Long Time Ago), respectively. These two items were highly correlated (*r* = .94) and were combined to create a measure of subjective time. Though the pattern of results was in the predicted direction (feels close condition: *M =* 3.49; *SD* = 2.75; feels far condition: *M* = 3.91 *SD* = 2.79) effects were not significant across conditions, *F*(1, 165) = 1.07, *p* = .302, which may be due to the different assessment used here compared to the original pilot study, which was designed to more closely mirror the experimental manipulation.

**Main Study (Reported in the Paper)**

**Exclusions**

We recruited a sample of 801 participants. We excluded 88 participants who reported they had not had sex with their partner in over 30 days, because one month was the range of the scale provided in the manipulation^[[1]](#footnote-1)^. Fourteen participants were excluded if their gender identity was not man or woman, given that gender was used as factor in analyses. Three participants were removed for failing our honest responder question. Three participants were removed for failing to meet our participant criteria (either being in their relationship for over a year, or not residing in the UK, the USA, or Canada).

**Other Measures and Analyses**

At the end of the study, participants were also asked how often they engaged in sexual activities with their partner (*M*=2.93, *SD*=1.17), as well as how often would they ideally engage in sexual activities with their partner (*M*=3.73, *SD*=1.42), both on scales ranging from 1="Not at all" to 8="More than once a day." We ran all analyses controlling for these sexual frequency and ideal sexual frequency items (in addition to controlling for objective days since sex, and quality of last sexual encounter), and all the effects remained consistent as when only controlling for objective days since sex.

In addition, the same measure of pro-relationship motivations in Study 1 (*α*=.95, *M*=5.88, *SD*=.89) was assessed post-manipulation. As an additional measure of motivation, participants also reported on their approach and avoidance relationship goals from 1=“Not at all” to 7=“Extremely” with items such as “I will be trying to deepen my relationships with my romantic partner” (approach goals: 4-items; *α*=.89, *M*=5.83, *SD*=.98) and “I will be trying to stay away from situations that could harm my romantic relationship” (avoidance goals 4-items; *α*=.61, *M*=5.30, *SD*=1.00) (adapted from Gable, 2006; Impett et al., 2008). There were no main effects of condition or gender or their interaction on pro-relationship motivations (subjective time *p*=.484; gender *p*=.632; 2-way interaction *p*=.299).

Results revealed no main effect of subjective time (*p*=.730) or gender (*p*=.929) on approach relationship goals. The interaction between gender and subjective time on relationship approach goals was significant, *F*(1,684)=5.00, *p*=.026, *η_p_^2^*=.007. Simple effect analyses, however, did not reveal a significant effect of subjective time on relationship approach goals for men (*p*=.067) or women (*p*=.186) separately. Results revealed no main effect of subjective time (*p*=.830) or gender (*p*=.777) on avoidance relationship goals. The interaction between gender and subjective time on relationship avoidance goals was significant, *F*(1,683)=4.99, *p*=.026, *η_p_^2^*=.007. Simple effect analyses, however, did not reveal a significant effect of subjective time on relationship avoidance goals for men (*p*=.082) or women (*p*=.158) separately. Finally, results revealed no main effects of condition (*p*=.892) or gender (*p*=.341), and no significant 2-way interaction between condition and gender (*p*=.241) on quality of last sexual encounter. However, the 2-way subjective time x gender interaction on relationship approach goals was reduced to non-significance, *F*(5,683)=3.68, *p*=.056, *η_p_^2^*=.005, when we controlled for quality of last sex.

**Study 3**

**Additional Outcome**

Each day, we asked participants about their *perception of their partner’s positive relationship behaviors*, using an 11-item version of the scale reported in Study 1 (adapted from Maxwell et al., 2019; *Rc*=.92, *M=*4.89, *SD*=1.59). In the analyses, we accounted for the number of days since sex, which was not associated with pro-relationship motivation, *b*=.01, *SE* =.01, *t=*.97, *p*=.331. When sex felt further away, people also perceived their partners as engaging in fewer pro-relationship behaviours (*b*=-.16, *SE* =.02, *t=*-10.03, *p*<.001), but there was no effect of partner’s perceptions (*b=*-.03, *SE* =.02, *t*=-1.67 *p=*.096). In the lagged day models, subjective time since sex today was associated with seeing a partner as less pro-relationship motivated tomorrow (*b*=-.05, *SE* =.02, *t=*-3.35, *p*<.001. And, tested the other way, on days when people saw their partner as more motivated, they felt like their last sexual experience was further away tomorrow (*b*=.34, *SE* =.05, *t=*7.186, *p*<.001).

**Follow-up Results**

Participants reported on their relationship satisfaction, sexual satisfaction and sexual desire in a baseline survey and a three-month follow-up survey. For relationship and sexual satisfaction, they completed the same measures as Study 1, and for sexual desire, they responded to two items adapted from the FSFI with both questions reverse coded so that higher scores indicate higher sexual desire (Rosen et al., 2000). After accounting for how satisfied participants were at the outset of the study (background sexual or relationship satisfaction, or sexual desire) as well as their sexual frequency over the three-week study, people who reported chronically feeling that sex was farther away (aggregate of subjective time since sex) reported feeling lower sexual, *b*=-.32, *SE* =.06, *t*(-5.63), *p*<.001, and relationship satisfaction, *b*=-.22, *SE* =.04, *t*(-5.46), *p*<.001, three months later, but no significant partner effects for relationship, *b*=-.07, *SE* =.04, *t*(-1.71), *p*=.088, or sexual satisfaction (*b*=-.03, *SE* =.05, *t*(-.56), *p*=.577). There were also no significant over time effects for own or partner sexual desire (own: *b*=-.01, *SE* =.04, *t*(.38), *p*=.701; partner: *b*=-.02, *SE* =.04, *t*(.60), *p*=.549.

**Auxiliary Analyses**

Gender (-1 = women; 1 = men) significantly moderated associations between subjective time since sex and both partners’ sexual satisfaction (own: *b* = -.064, *SE* =.02, *t*(-4.02), *p* < .001, partner: *b* = .042 *SE* =.02, *t*(2.64), *p* = .008), and both a person’s perceptions of their partner’s pro-relationship behaviors (*b* = -0.61, *SE* =.02, *t*(-3.39), *p* < .001) and their partner’s perceptions of them (*b* = -.06, *SE* =.02, *t*(3.17), *p* =.002). When both men and women felt that the last sexual encounter was further away, they reported lower sexual satisfaction (men: *b* = -.20, *SE* =.01, *t*(-13.90), *p* < .001; women: *b* = -.10, *SE* =.01, *t*(-8.53), *p* < .001) and perceived less pro-relationship behaviors (men: *b* = -.17, *SE* =.02, *t*(-10.58), *p* < .001; women: *b* = -.11, *SE* =.01, *t*(-7.57), *p* < .001), but these associations were strongest for men. In addition, women saw their partner as less pro-relationship motivated when their partner reported that the last sexual experience felt farther away (*b* = -.07, *SE* =.01, *t*(-5.05), *p* < .001), but there was no effect of a partner’s subjective feelings of time since sex for men (*b* = -.02, *SE* =.02, *t*(-1.58), *p* = .113). For the overtime associations, the association between subjective time since sex over the course of the diary and sexual satisfaction three months later was moderated by gender (*b* = -.14, *SE* =.06, *t*(-2.54), *p* = .012). Chronic feelings over the diary study that sex was farther away were linked to lower sexual satisfaction three months later for both men and women (men: *b* = -.54, *SE* =.09, *t*(-6.09), *p* < .001; women: *b* = -.17 *SE* = .08, *t*(-2.25), *p* = .026), though this effect was stronger for men.

1. This exclusion was not mentioned in the pre-registration but was done ahead of analyses, since it did not make conceptual sense to have participants included in the study who did not have sex within the endpoints of the scale. [↑](#footnote-ref-1)
